# Transcranial Ultrasound Stimulation as an Emerging Treatment for Substance use Disorder: Promise and Precaution

**DOI:** 10.1007/s40429-026-00734-2

**Published:** 2026-04-30

**Authors:** Mica Komarnyckyj, Nima Norbu Sherpa, Will Lawn, Elsa Fouragnan

**Affiliations:** 1 Manchester Biomedical Research Centre, Division of Psychology & Mental Health, University of Manchester, Oxford Rd, Manchester, M13 9PL UK; 2 School of Psychology, Faculty of Health, University of Plymouth, Plymouth, UK; 3 Brain Research Imaging Center, Faculty of Health, University of Plymouth, Plymouth, UK; 4 Department of Psychology, Institute of Psychiatry, Psychology & Neuroscience, King’s College London, London, UK

## Introduction

Neuromodulation has gained attention as an alternative non-pharmacological approach for treating substance use disorders (SUDs). Non-invasive brain stimulation methods, particularly those targeting cortical regions which are part of wider reward-processing networks, have been widely investigated, with repetitive transcranial magnetic stimulation (rTMS) showing modest reductions in craving [1].

Dysfunction of subcortical regions within these reward networks, such as the ventral striatum and ventral tegmental area, however, are thought to play a more significant role in the development and maintenance of SUDs [2]. These deeper brain regions have historically been inaccessible by non-invasive neuromodulation and could only be reached with deep brain stimulation (DBS) which requires surgery to the skull and brain [1, 3].

Transcranial ultrasound stimulation (TUS) – also referred to as low-intensity transcranial focused ultrasound stimulation (LIFU or tFUS) – is an emerging non-invasive neuromodulation method under investigation for SUD treatment [3] and broader psychiatry applications [4]. Animal and human studies show that TUS can safely modulate subcortical regions with millimetre-scale precision (1–5 mm), including the nucleus accumbens (NAcc) within the ventral striatum which is a central part of the reward network [5–8].

TUS therefore holds promise for alleviating symptoms associated with dysfunctional reward processing, such as addictive behaviours, compulsivity and anhedonia. This is a step-change compared with established non-invasive methods – rTMS and transcranial direct current stimulation (tCDS) – which are limited to cortical and centimetre-scale brain targets [1, 7]. The unprecedented ability to stimulate deep in the brain, however, brings a new set of scientific and ethical challenges.

## What is Low-intensity Transcranial Ultrasound Stimulation?

Focused ultrasound stimulation encompasses low-, intermediate-, and high-intensity regimes, each with distinct uses and safety profiles. High-intensity ultrasound aims to produce irreversible structural changes through thermoablation. FDA-approved and CE-marked uses of high-intensity ultrasound include transcranial thalamotomy for treatment of essential tremor and Parkinson’s motor problems, as well as ablative treatments outside of the brain for prostate tumours, uterine fibroids and osteoid osteoma. Intermediate-intensity ultrasound can induce reversible effects that extend beyond normal physiology, for example microbubble mediated blood brain barrier opening under stable cavitation [9].

**Fig. 1:**
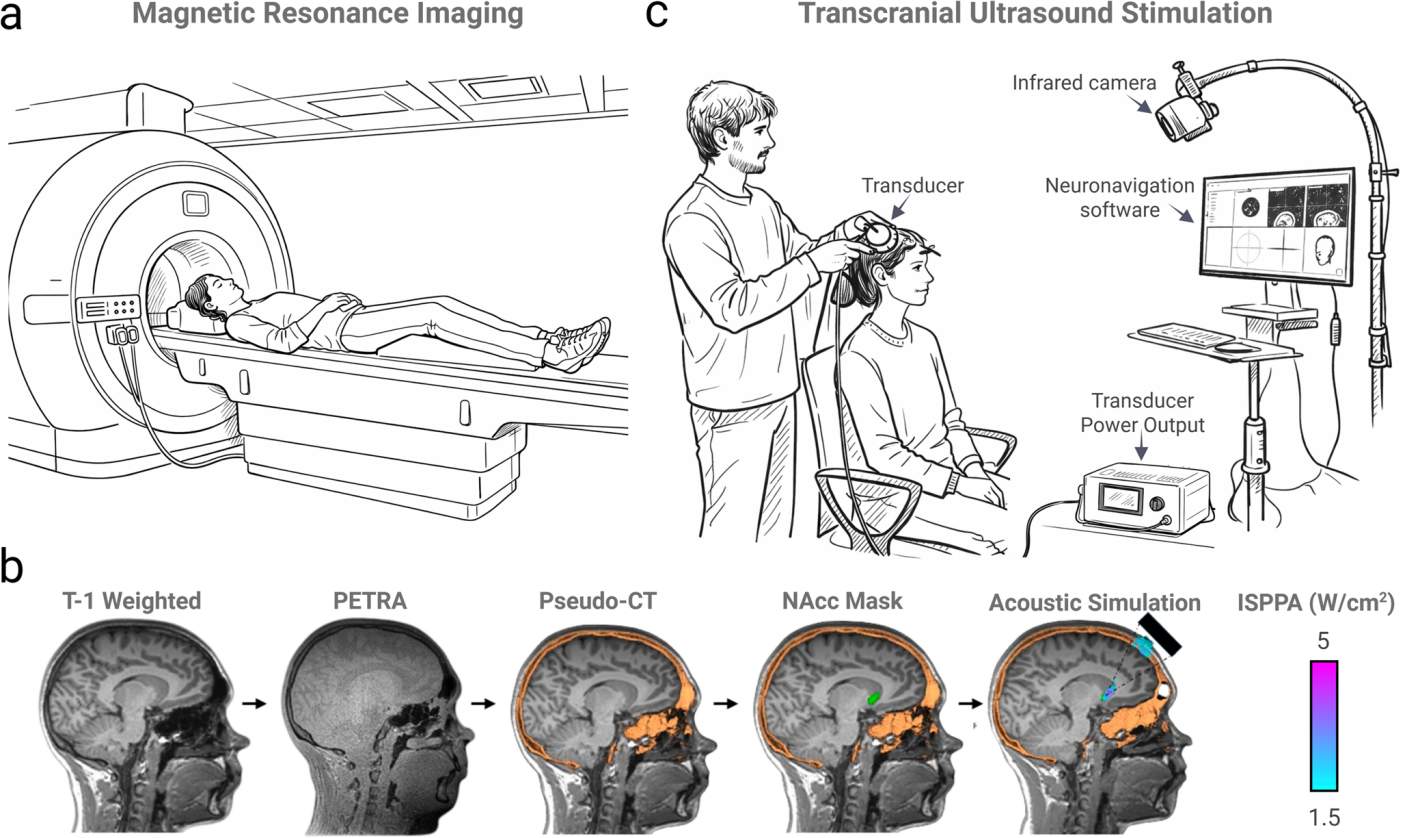
Example workflow for Transcranial Ultrasound Stimulation. (**a**) Prior to neurostimulation a T1-weighted, T2 weighted and ultra-short echo-time images may be acquired in a magnetic resonance imaging (MRI) scanner for individual brain and skull anatomy. (**b**) MRI images are co-registered and ultra-short echo-time may be converted into a pseudo-CT to estimate skull density and geometry, facilitating personalised modelling. Acoustic and thermal simulations are performed to model ultrasound transmission, enabling optimisation of stimulation parameters (e.g., frequency, duration, location) whilst ensuring International Transcranial Ultrasonic Stimulation Safety and Standards Consortium guidelines for safety are adhered to. Abbreviations: PETRA (Pointwise Encoding Time reduction with Radial Acquisition), ISPPA (Spatial-Peak Pulse-Average). (**c**) During the transcranial ultrasound stimulation (TUS) procedure, a handheld transducer generates low-intensity ultrasound waves (typically between 300 kHz and 1 MHz) that are focussed into a narrow, typically elliptical beam a few millimetres in size. First, a degassed ultrasound gel is applied to the hair, to allow transmission of the acoustic energy. The transducer is then coupled to the scalp with gel, aiming to create an air free pathway for ultrasound transmission. Neuronavigation software may guide the beam in real-time to the pre-defined brain target, allowing precise and personalised modulation of the desired circuits. Please note that this is one of many possible configurations for TUS

In contrast, low-intensity ultrasound (i.e., TUS) induces fully reversible effects whilst preventing excessive heating, tissue damage or cavitation. Converging evidence suggests modulation of neural activity is driven by the mechanical effects of acoustic waves, including transient displacement of neuronal membranes and activation of mechanosensitive ion channels [7, 9, 10]. Low-intensity ultrasound not yet received FDA approval or CE marking for transcranial use in the treatment of psychiatric and neurological conditions. This perspective focuses exclusively on low-intensity regimes that remain under the safety limits highlighted by the International Transcranial Ultrasonic Stimulation Safety and Standards Consortium (ITRUSST) [9]. Figure 1 illustrates an example workflow for precise and safe TUS which is tailored to individual neuroanatomy.

## Why is TUS Promising for Substance use Disorder Treatment?

SUD remains one of the most entrenched challenges in behavioural and mental health. It is a relapsing, chronic condition, in which people experience harm from persistent substance use and repeatedly try to change their behaviour but struggle to stop. Despite pharmacological and psychosocial treatment development, people with SUD continue to show high relapse rates with limited long-term efficacy of treatments [2]. Invasive neuromodulation of the NAcc with DBS has delivered encouraging reductions in craving and consumption in SUD; however, relapse is common with this treatment and the for surgical electrode implantation imposes clear limitations on scalability and accessibility [11].

In contrast, TUS does not require invasive neurosurgery and can reach the same subcortical targets as DBS [3–6, 8, 12]. Compared with established non-invasive methods, TUS has a uniquely broad theoretical capacity to interface with the brain: it can target multiple sites simultaneously [10], operate in a holographic manner [13] or achieve precise perturbations such as single or double disconnections [12, 14]. These features help explain the recent surge of interest in this technology [15] and its promise for SUD research and treatment.

By combining TUS with neuroimaging, researchers can explore its effects on brain activity - either online (during neurostimulation) or offline (after neurostimulation). This enables non-invasive evaluation of neurocircuitry theories of SUD, including subcortical regions and cognitive processes. For example, the function of the mesolimbic dopamine pathway in reward processes that drive drug-seeking (incentive salience and craving); the amygdala contribution in negative emotional states that contribute to relapse; and ventromedial prefrontal cortex involvement in drug valuation and decision-making [16, 17]. Moreover, TUS can be applied to multiple regions simultaneously [10], either synchronously or asynchronously, allowing for more complex network-level theories to be evaluated and providing avenues for treatments with multiple brain targets.

These applications are highly relevant given the heterogeneity of SUDs, where similar symptom profiles may emerge from distinct network-level dysfunction. Instead of assuming one-size-fits-all targets, in the long-term TUS may support a shift toward precision neuroscience [18] and computationally informed interventions [19, 20]. This lays the groundwork for personalised and adaptive interventions. Depending on further progress in neurofeedback technologies, this could include closed-loop approaches, where TUS is adjusted based on ongoing brain signals to support self-regulation of SUD symptoms, such as craving or negative emotions [21].

SUD is a biopsychosocial disorder and cannot solely be attributed to disrupted brain mechanisms. Risk for SUD and individual treatment response are also shaped by non-biological factors, such as experiences of trauma, economic disadvantage and interpersonal relationships [22, 23]. Neuromodulation alone is therefore unlikely to provide long-lasting symptom relief for most people without effective integration with psychosocial therapies [11]. TUS should not be viewed as a silver bullet, but instead a tool that can substantially contribute to circuit-level insight for SUD and precision psychiatry. For those with severe SUD who have not responded to standard treatments, ongoing modulation of deep brain regions that underpin craving, incentive salience and habit, may be a valuable additional line of treatment for supporting abstinence.

## What are the TUS Biophysical Safety Limits?

Drawing on expert consensus, biomedical device regulations and broader ultrasound literature, the ITRUSST have defined biophysical safety limits [9] and reporting recommendations [24] for low-intensity ultrasound stimulation. Given the rapid expansion in TUS as a research and clinical tool for a wide range of neurological and psychological disorders [15], it is imperative that the research community disseminates and adheres to these guidelines [9, 24].

Critically, ITRUSST defines operational boundaries to ensure TUS exposure is within nonsignificant biophysical risk thus preventing irreversible brain changes [9]. TUS protocols should maintain the mechanical index (MI) or mechanical index for transcranial application (MItc) at or below 1.9 to minimise the risk of cavitation. Additional limits are also defined to minimise tissue heating and prevent thermal damage. Only when operating under these limits can TUS be regarded as posing nonsignificant risk and therefore be classed as non-invasive and low-intensity [9, 24]. Serious adverse effects have not been reported when operating within these nonsignificant risk limits, however, mild to moderate reversible side effects may include headache, neck pain, muscle twitches, anxiety, problems with attention, nausea and vomiting [25, 26].

Operating outside of these limits exposes participants to increased risk of adverse bioeffects [27]. Focused ultrasound can penetrate deep brain regions and exert both mechanical and thermal action on tissue. At very high negative peak pressure amplitudes, inertial cavitation can occur, which may cause brain haemorrhage, cell death and permanent changes in the structure and function of a region or network [28]. Risk is elevated by significant variability of both human skull anatomy and TUS equipment, ranging from single-element handheld devices to complex multi-element arrays with MRI-guided targeting. Differences in transducer size, frequency, pulse parameters, and skull-correction methods result in variable acoustic exposure and bioeffects across studies.

An important open question is whether overdose-related brain injury, specific to drugs which can cause respiratory depression (e.g., opiates, alcohol and benzodiazepines), increases vulnerability to adverse bioeffects of TUS. During an overdose there may be insufficient oxygen supply to the brain (i.e., hypoxia) which can damage brain microstructure [29]. Potentially, this could alter nonsignificant risk thresholds or pressure required to cause inertial cavitation which have been defined based on healthy individuals who are not at high risk to adverse bioeffects due to vascular vulnerabilities or pre-existing brain damage [9]. For safe translation of TUS into SUD treatment, dedicated studies will be needed to define these risk thresholds. Investigating the interplay between overdose-related brain vulnerability and TUS biophysical safety, therefore, represents a critical gap for future research.

Concerningly, at present, adherence with ITRUSST recommendations [9, 24] varies across countries and institutions, and compliance is not formally mandated. The importance of adhering to biophysical safety limits is, however, underscored by a recent serious adverse event during a focussed ultrasound trial for opioid use disorder (NCT06218706). This trial documents brain injury, including microhaemorrhages and the observed structural changes suggest cavitation occurred [30]. Retrospective analysis showed ultrasound exposure significantly exceeded the nonsignificant risk threshold (MItc > 1.9) [27, 31, 32].

A subsequent open letter concluded that the protocol cannot be classified as low-intensity but instead represents a high-intensity regime based on the high mechanical index and irreversible brain injury [31]. Open label feasibility trials conducted by the same research group, using equivalent intensity focussed ultrasound regimes, have targeted the NAcc as a treatment for opioid and co-occurring SUDs [33, 34]. As these were not conducted within the low-intensity regime, they fall outside the scope of this article.

Future researchers operating outside of the low-intensity (i.e., non-invasive) safety limits should avoid this terminology. Instead, protocols should be accurately classified according to the expert-defined exposure regimes (high-, intermediate-, or low-intensity) that reflect both intended effects on brain structure (i.e., reversible vs. irreversible changes) and true risk [9].

## What Lies Ahead for TUS Within SUD Treatment?

Research with low-intensity TUS is rapidly expanding [15]; however, TUS is not yet approved for psychiatric indications and has not been widely evaluated for SUD treatment. Due to the chronic and relapsing nature of SUD, sustained therapeutic benefit will likely require repeated stimulation. This raises important questions about the long-term effects of targeting important deep brain regions of the addiction and reward circuitry (NAcc) [5, 6, 8]. Repeated stimulation of focal regions, such as the NAcc, is expected to induce localised neurophysiological changes; however, it may also affect reward sensitivity and connectivity.

The NAcc is involved in many other behavioural and vital life processes – motivation, decision-making, learning, pain, and emotional processing – that TUS may impact. Whether such changes enhance, preserve, or inadvertently disrupt circuit function remains unknown. Given that altered reward processing is a defining feature of many SUDs, any further manipulation of these circuits must be approached with caution and long-term follow-up. Trials are needed to establish efficacy, define optimal dosing regimens, and monitor for both therapeutic and unintended long-term effects.

To uncover the breadth of planned research evaluating TUS for SUD treatment, our team conducted a review of global clinical trial databases on 11th December 2025 including ClinicalTrials.gov, clinicaltrialsregister.eu and www.isrctn.com (Supplementary Table 1). ClinicalTrials.gov was the only database that contained relevant trials with both a TUS intervention and SUD as the targeted health condition, with eleven pre-registered trials meeting these criteria. There was a dominance of US studies (*n* = 9), with a single study in China and the UK. Planned sample sizes range from 10 to 126 participants including early stage open-label single group studies, up to more robust double-blind placebo-controlled randomised trials (Table 1).

**Table 1:** Characteristics of pre-registered trials investigating transcranial ultrasound stimulation for the treatment of substance use disorders. A targeted search of ClinicalTrials.gov, clinicaltrialsregister.eu and www.isrctn.com search identified 11 pre-registered studies investigating Transcranial Ultrasound Stimulation (TUS) interventions for substance use disorders. Trials characteristics such as design, brain region targeted estimated completion and estimated sample size are included in this table. Abbreviations: tDCS (Transcranial direct current stimulation), EFT (episodic future thinking)

Principal investigator	Trial ID	Sponsor (Location)	Substance	Study design	Brain region	Intervention	Estimated completion (Status)	Randomisation / Masking	Target sample size
J. Du	NCT06867224	Shanghai Mental Health Center (China)	Alcohol	Randomised, triple-blind, parallel-group	Ventromedial prefrontal cortex	TUS	01/01/2027 (Not yet recruiting)	Yes / Triple	40 participants
E.F. Fouragnan	NCT06894966	University of Plymouth (UK)	Alcohol	Within-subject, randomised, double-blind, sham-controlled	Not defined	TUS	20/09/2026 (Not yet recruiting)	Yes / Triple	30 participants
S. LaConte	NCT05901610	Virginia Polytechnic Institute & State University (USA)	Alcohol	Non-randomised, single-blind, crossover	Insular cortex	EFT + TUS	31/12/2025 (Suspended)	No / Single	10 participants
K. Moussawi	NCT06518785	University of California (USA)	Alcohol	Double-blind, controlled, randomised, complete block, 2-period crossover	Basal ganglia and thalamus	TUS	09/2030 (Recruiting)	Yes / Double	25 participants
N. Ait-Daoud Tiouririne	NCT05857852	University of Virginia (USA)	Cocaine	Randomised, single-blind, crossover	Dorsal anterior insula	TUS	01/03/2025 (Unknown)	Yes / Triple	30 participants
M. Phillips	NCT06477029	University of Pittsburgh, USA	Cocaine	Randomised, single-blind, crossover	Ventral striatum	TUS	15/03/2026 (Recruiting)	Yes / Single	31 participants
C.R. Estebanez	NCT04379115	Case Western Reserve University USA)	Opioid	Double-blind, placebo controlled, randomised	Not defined	TUS (Active/Sham) + tDCS	31/10/2027 (Recruiting)	Yes / Double	126 participants
J. Florig	NCT06297200	Virginia Polytechnic Institute & State University (USA)	Opioid	Open-label, single-group, within-subject sham controlled	Not defined	TUS	10/2026 (Recruiting)	No / None (open label)	25 participants
M.R. Lee	NCT06453109	Washington D.C. Veterans Affairs Medical Center (USA)	Opioid	Within subject, randomised, double-blind, sham-controlled	Anterior insula	TUS	30/09/2025 (Recruiting)	Yes / Double	25 participants
M. Ranjan	NCT07010016	West Virginia University (USA)	Opioid & substance use disorders	Open-label, single-group	Nucleus accumbens and ventral capsule	TUS	30/07/2030 (Not yet recruiting)	No / None (open label)	20 participants
M. R. Lee	NCT06405074	Washington D.C. Veterans Affairs Medical Center (USA)	Tobacco	Within subject, randomised, double-blind, sham-controlled	Dorsal anterior insula	TUS	31/03/2026 (Recruiting)	Yes / Double	44 participants

Brain regions within the reward network were common targets in pre-registered trials, including the ventral striatum and its subregion, the NAcc (*n* = 3); the ventromedial prefrontal cortex (*n* = 1) which connects the ventral striatum to higher-order prefrontal control systems; and the thalamus and basal ganglia circuitry (*n* = 1) which supports decision-making, motivation and habit formation [35, 36]. The insular cortex (including the anterior insula) was targeted by several studies (*n* = 4), a region interconnected with amygdala-striatal networks that is theorised to contribute to craving, drug-seeking, decision-making and withdrawal [37]. Notably, three studies did not define the brain regions targeted.

Opioid (*n* = 4) and alcohol use disorders (*n* = 4) represent the most frequent clinical targets, followed by cocaine (*n* = 2). There was only one study investigating tobacco use disorder, a limited number given that tobacco addiction causes most deaths and public health harm, by far. Despite the rise in problematic cannabis use particularly among young people, there is also a total absence of studies evaluating TUS for this substance [38].

Together, these registrations illustrate how TUS is being positioned within SUD research. Given the limited sample sizes, most pre-registered studies will have insufficient power to detect clinically meaningful effects or give definitive causal evidence. These studies will, however, play an important role in gaining mechanistic insight, establishing feasibility and tolerability, and generating preliminary evidence which can be used to inform and justify investment in larger-scale trials.

## Conclusion

TUS represents a leap forward in our ability to manipulate brain activity, with precision and at depth, to test causal brain circuits implicated in craving and compulsive behaviour, making it an exciting candidate for better understanding the neural basis of SUD. In the longer-term, these initial mechanistic studies will hopefully lead to larger clinical trials and ultimately modulate previously inaccessible deep brain regions, which underpin addictive behaviours, to support relapse prevention. At this stage, the priority is clear for researchers. We must adhere to experts’ safety and reporting guidelines to ensure participant safety and transparently share lessons to guide the safe advancement of TUS in SUD treatment.

## Key References

- Aubry JF, Attali D, Schafer ME, Fouragnan E, Caskey CF, Chen R, et al. ITRUSST consensus on biophysical safety for transcranial ultrasound stimulation. Brain Stimul. 2025;18(6):1896-905. 10.1016/j.brs.2025.10.007.
  - ◦ Expert consensus defined nonsignificant risk levels of low-intensity regime TUS based on mechanical and thermal effects.
- Attali D, Daniel M, Plaze M, Aubry JF. Strengths and weaknesses of transcranial ultrasound stimulation and its promise in psychiatry: an overview of the technology and a systematic review of the clinical applications. Int J Hyperthermia. 2025;42(1):2539986. 10.1080/02656736.2025.2539986.
  - ◦ Systematic review of studies which applied TUS in clinical applications including 10 human studies covering depression, anxiety, schizophrenia and substance use disorder.
- Yaakub SN, Eraifej J, Bault N, Lojkiewiez M, Bellec E, Roberts J, et al. Non-invasive ultrasonic neuromodulation of the human nucleus accumbens impacts reward sensitivity. Nat Commun. 2025;16(1):10192. 10.1038/s41467-025-65080-9.
  - ◦ TUS targetng the NAcc altered BOLD responses to reward signals and reward learning behaviour (26 healthy human participants).

## Supplementary Information

Below is the link to the electronic supplementary material.

Supplementary Material 1 (DOCX 21.6 KB)

## Data Availability

No datasets were generated or analysed during the current study.
