# Supplementary material for: Transcranial Ultrasound Stimulation as an Emerging Treatment for Substance use Disorder: Promise and Precaution
Source: Curr Addict Rep. 2026 Apr 30;13(1):45. doi: 10.1007/s40429-026-00734-2 (PMC13133219; doi:10.1007/s40429-026-00734-2)
Supplement: Supplementary file 1 — Supplementary Material 1 (DOCX 21.6 KB) [file 40429_2026_734_MOESM1_ESM.docx]

**Supplementary Material**

**Transcranial ultrasound stimulation as an emerging treatment for substance use disorder: Promise and Precaution**

Mica Komarnyckyj^1^, Nima Norbu Sherpa^2,3,4^, Will Lawn^4^, Elsa Fouragnan^2,3^

^1^ Biomedical Research Centre, Division of Psychology & Mental Health, University of Manchester, Oxford Rd, Manchester, M13 9PL

^2^ School of Psychology, Faculty of Health, University of Plymouth, Plymouth, UK

^3^ Brain Research Imaging Centre, Faculty of Health, University of Plymouth, Plymouth, UK

^4^Department of Psychology, Institute of Psychiatry, Psychology & Neuroscience, King's College London, London, UK

*Corresponding authors: mica.komarnyckyj@manchester.ac.uk and elsa.fouragnan@plymouth.ac.uk

**Supplementary Table 1. Preregistered trial review methodology including search strategy and relevant trials identified in each clinical trial database**

The preregistered trial review included three trial databases (ClinicalTrials.gov, clinicaltrialsregister.eu and [www.isrctn.com](http://www.isrctn.com)) each with unique search strategies based on their formatting requirements which are given in this table. The review uncovered 29 potentially relevant trials, which were independently screened by two reviewers (M. Komarnyckyj and N. Sherpa) to identify 11 trials which met the inclusion criteria (INCLUDE: “TUS intervention” AND “substance use disorder/addiction”).

| **U.S. National Library of Medicine (N = 28, N included = 11)**  [**clinicaltrials.gov**](https://clinicaltrials.gov/) | | | | |
| --- | --- | --- | --- | --- |
| Search strategy = #1 AND #2 | | | | |
| #1 | ("substance use disorder" OR "substance-use disorder" OR addiction OR "addictive behavior" OR "addictive disorder" OR alcohol* OR opioid* OR methamphet* OR cocaine* OR cannab* OR amphetamin* OR nicotine* OR tobacco*) | | | |
| #2 | ("low intensity focused ultrasound" OR "low-intensity focused ultrasound" OR "low intensity focused ultrasound stimulation" OR "low-intensity focused ultrasound stimulation" OR "low intensity pulsed ultrasound" OR "low-intensity pulsed ultrasound" OR "focused ultrasound neuromodulation" OR "transcranial focused ultrasound stimulation" OR "transcranial focused ultrasound" OR "transcranial ultrasound stimulation" OR "magnetic resonance-guided focused ultrasound" OR "transcranial pulse stimulation" OR LIFU OR tFUS) | | | |
| **EU Clinical Trials Register (EUCTR) (N = 0)**  **www.clinicaltrialsregister.eu**  Search strategy = #1 AND #2 | | | | |
| #1 | ("substance use disorder" OR "substance-use disorder" OR addiction OR "addictive behavior" OR "addictive disorder" OR alcohol* OR opioid* OR methamphet* OR cocaine* OR cannab* OR amphetamin* OR nicotine* OR tobacco*) | | | |
| #2 | ("low intensity focused ultrasound" OR "low-intensity focused ultrasound"  OR "low intensity focused ultrasound stimulation" OR "low-intensity focused ultrasound stimulation" OR "low intensity pulsed ultrasound" OR "low-intensity pulsed ultrasound" OR "focused ultrasound neuromodulation" OR "transcranial focused ultrasound stimulation" OR "transcranial focused ultrasound" OR "transcranial ultrasound stimulation" OR "magnetic resonance-guided focused ultrasound" OR "transcranial pulse stimulation" OR LIFU OR tFUS) | | | |
| **ISRCTN, The UK’s Clinical Study Registry (N = 1, N included = 0)**  [**www.isrctn.com**](http://www.isrctn.com) | | | | |
| Search strategy = Intervention AND Health condition | | | | |
| Search | Intervention | Health condition | N | N included |
| 1 | ultrasound | substance use disorder | 0 | 0 |
| 2 | ultrasound | substance-use disorder | 0 | 0 |
| 3 | ultrasound | addiction | 0 | 0 |
| 4 | ultrasound | alcohol | 1 | 0 |
| 5 | ultrasound | opioid | 0 | 0 |
| 6 | ultrasound | methamphetamine | 0 | 0 |
| 7 | ultrasound | cannabis | 0 | 0 |
| 8 | ultrasound | cocaine | 0 | 0 |
| 9 | ultrasound | amphetamine | 0 | 0 |
| 10 | ultrasound | nicotine | 0 | 0 |
| 11 | ultrasound | tobacco | 0 | 0 |
